# Achilles enthesitis on physical examination leads to worse outcomes after 2 years of follow up in patients with ankylosing spondylitis from REGISPONSER-AS registry

**DOI:** 10.1186/s13075-023-02988-x

**Published:** 2023-01-13

**Authors:** Clementina López-Medina, M. Ángeles Puche-Larrubia, Raquel Granados, Lourdes Ladehesa-Pineda, Desirée Ruiz-Vilchez, M. Carmen Ábalos-Aguilera, Pilar Font-Ugalde, Eduardo Collantes-Estévez

**Affiliations:** 1 Department of Rheumatology, Reina Sofia University Hospital, Avda. Menendez Pidal, S/N. 14004, Cordoba, Spain; 2 Maimonides Biomedical Research Institute of Cordoba (IMIBIC), Cordoba, Spain; 3 Medical and Surgical Sciences Department, University of Cordoba, Cordoba, Spain

**Keywords:** Ankylosing spondylitis, Enthesitis, Outcomes

## Abstract

**Background:**

Enthesitis represents one of the most important peripheral musculoskeletal manifestations in patients with axial spondyloarthritis (axSpA). However, studies specifically evaluating Achilles tendon enthesitis and its impact over time are scarce. The objectives of this study were to evaluate the impact of Achilles’ tendon enthesitis found at baseline during physical examination on the outcome measures after 2 years of follow-up in patients with ankylosing spondylitis (AS).

**Methods:**

This was an observational and prospective study conducted during 2 years of follow-up in the REGISPONSER-AS registry. Linear regression models adjusted for age, body mass index (BMI), and anti-TNF intake were conducted to evaluate the association between the presence of Achilles enthesitis at baseline and the patient-reported outcome (PRO) scores at baseline. The impact of this feature on PROs over 2 years of follow-up was evaluated using mixed models for repeated measures adjusted for age, BMI, and anti-TNF intake.

**Results:**

Among the 749 patients included, 46 patients (6.1%) showed Achilles’ tendon enthesitis during physical examination at the baseline study visit. Patients with Achilles enthesitis had an increase in the global VAS score, BASDAI, mBASDAI, ASDAS-CRP, and BASFI scores in comparison with patients without this feature. In addition, the mean global VAS, BASDAI, and ASDAS-CRP scores were significantly higher among patients with Achilles enthesitis over the 2 years of follow-up after adjusting for age, BMI, and current anti-TNF intake. The percentage of patients achieving ASDAS low disease activity (ASDAS < 2.1) after 2 years of follow-up was 15.9% and 31.5% for patients with and without Achilles enthesitis, respectively (*p* = 0.030).

**Conclusions:**

In patients with AS, the presence of Achilles’ tendon enthesitis was associated with worse scores on the outcome measures after 2 years of follow-up, leading to a lower probability of achieving low disease activity.

## Background

Axial spondyloarthritis (axSpA) is a chronic inflammatory rheumatic disease that mainly affects the axial skeleton (spine and sacroiliac joints) and includes both patients with structural damage on X-ray (also known as radiographic axial SpA or ankylosing spondylitis (AS)) and patients without definitive signs of structural damage (nonradiographic axSpA) [1]. Apart from axial involvement, patients with axSpA may suffer from peripheral musculoskeletal manifestations such as enthesitis, arthritis and dactylitis.

Enthesitis is considered a pathological hallmark of SpA, representing inflammation of the insertion of tendons, ligaments, aponeuroses, and capsules into the bone [2]. Approximately 30–40% of patients with axSpA will suffer from peripheral enthesitis at some point during the course of the disease [3, 4], with the heel (Achilles’ tendon and plantar fascia insertions) being the most frequently affected location [5, 6]. In fact, heel enthesitis is included in the Amor and ASAS (Assessment of Spondyloarthritis International Society) classification criteria as a feature to classify patients with axSpA [1, 7].

Factors associated with peripheral enthesitis include the presence of peripheral arthritis, dactylitis and psoriasis [3–6]. In addition, observational studies suggest that enthesitis is associated with a higher burden of disease in comparison with patients who do not have enthesitis. A recent publication in the DESIR cohort (that included patients with recent onset axSpA) demonstrated that participants with peripheral enthesitis showed more severe disease activity and worse functional outcomes, highlighting the impact of enthesitis on the burden of the disease [6]. Similarly, another study showed an association between the Mander Enthesitis Index (MEI) and morning stiffness, disease duration, and quality of life [8]. However, these previous studies evaluated enthesitis using different indices without discriminating the specific anatomical locations, which prevented the assessment of the impact of such locations on the outcome measures. In addition, these studies considered patients with enthesitis to have suffered from this feature at any time during the course of the disease, without differentiating patients with enthesitis at the time of the study visit from those with a past history of enthesitis.

We consider it important to analyze the profile of patients who will have enthesitis during a consultation in daily practice and to predict the course of these patients over time. For these reasons, we decided to conduct this study, with the aim of evaluating the profile of patients with Achilles’ tendon enthesitis (found during physical examination) and its impact on the outcome measures after 2 years of follow-up in patients with AS (or r-axSpA).

## Methods

### Design and patients

REGISPONSER-AS is an observational, longitudinal, and prospective study including a subgroup of 749 patients fulfilling the modified New York criteria for radiographic sacroiliitis from the national REGISPONSER study (Spondyloarthritis Registry of the Spanish Rheumatology). The original REGISPONSER registry is a multicenter Spanish study that incorporated patients with SpA who fulfilled the European Spondyloarthropathy Study Group (ESSG) criteria for SpA [9] between March 2004 and March 2007. The design, sampling, and recruitment of the patients in the registry have been previously described [10].

A total of 2367 patients were consecutively included in REGISPONSER, and each patient was assigned a random code in the database. Patients randomly sampled from the original REGISPONSER registry were included in the REGISPONSER-AS prospective study if they fulfilled the following inclusion criteria: (A) confirmed cases of AS as defined by the modified New York criteria [11]; (B) blood tests available within 15 days of the visit and a complete radiographic study within the previous year; and (C) agreement to complete all self-administered questionnaires. The total follow-up period of REGISPONSER-AS was 5 years with annual visits, although only the first 2 years of follow-up were considered in the present study, in which data were collected at baseline and at the 1-year and 2-year time points.

This study was approved by the Ethics Committee (“Comisión de Ética e Investigación Sanitarias”) from the Reina Sofia University Hospital of Córdoba (Spain) on 21 April 2006, and all of the participants signed an informed consent form to participate in the REGISPONSER registry.

### Collected variables

A case-report form was used to collect clinical data during a face-to-face meeting at each study visit. Information concerning clinical events that occurred before the baseline study visit were collected retrospectively by asking the patients or checking their medical records. This study included the following variables:

- Sociodemographic Data: sex, age, body mass index (BMI), university education, marital status, and smoking status.
- Clinical characteristics: Age of onset of SpA, disease duration (years between symptom onset and the baseline study visit), diagnostic delay (years between symptom onset and the SpA diagnosis), family history of SpA, HLA-B27 antigen status, C-reactive protein (CRP, mg/dL), synovitis ever, psoriasis, inflammatory bowel disease (IBD), enthesitis ever, dactylitis, and uveitis. A physical examination was conducted at each visit to evaluate the number of swollen joints and the number of painful entheses according to the Maastricht Ankylosing Spondylitis Score (MASES) [12]. In addition, the specific locations of any enthesitis during the physical examination were collected (including Achilles’ tendon enthesitis, defined as tenderness after pressure at the site of the insertion of the Achilles tendon on the calcaneus).
- Patient-reported outcomes (PROs): Disease activity at each study visit was evaluated using the Bath Ankylosing Spondylitis Disease Activity Index (BASDAI) [13], modified BASDAI (mBASDAI, which omits questions 3 and 4) [14], the patient’s global visual analog scale (global VAS) [15] and the Ankylosing Spondylitis Disease Activity Score (ASDAS) [16], while function was evaluated using the Bath Ankylosing Spondylitis Functional Index (BASFI) [17]. The Mental Health Survey (MSF12) and the Physical Health Survey (FSF12) from the SF12 questionnaire were completed by the participants [18].
- Structural damage: The total Bath Ankylosing Spondylitis Radiology Index (BASRI) was evaluated locally to determine the structural damage in the spine [19].
- Treatment: Data on previous and current treatments were collected, such as the use of nonsteroidal anti-inflammatory drugs (NSAIDs), conventional synthetic disease-modifying antirheumatic drugs (csDMARDs: sulfasalazine, methotrexate or leflunomide), and biological DMARDs (anti-TNF treatment).

Prior to the initiation of the study, participating researchers undertook a 1-day training course in clinical examination techniques to avoid variability among investigators.

### Statistical analysis

Descriptive data are shown as the mean and standard deviation (SD) for quantitative variables and as absolute and relative frequencies for qualitative variables.

According to the presence of Achilles’ tendon enthesitis during the physical examination at the baseline visit, patients were divided into “current Achilles enthesitis” vs. “no current Achilles enthesitis.” Baseline clinical characteristics, PROs, and treatments were compared across the two groups using chi-squared tests and *t* tests for binary and continuous variables, respectively.

Baseline data were used to evaluate whether the presence of current Achilles enthesitis influenced the PROs (*β* coefficient). Linear regression models were conducted using the PROs as the dependent variable and the presence of Achilles enthesitis during the physical examination as the explanatory variable. Since age, BMI, and the current use of anti-TNF therapy may influence both the presence of enthesitis and the PROs, additional models adjusted for these variables were explored.

After that, longitudinal data were used to evaluate the impact of baseline current Achilles enthesitis on PROs over 2 years of follow-up (i.e., considering all of the time points) using mixed models for repeated measures (MMRM), using age, BMI and anti-TNF intake as fixed effects and the patient as a random effect.

Finally, the cumulative percentage of patients achieving ASDAS low disease activity (ASDAS < 2.1) and remission, defined as ASDAS inactive disease (ASDAS < 1.3), after 2 years of follow-up were compared between the two groups using the chi-square test.

All contrasts were bilateral and considered significant with a *p* value < 0.05. Data were collected, processed and analyzed using RStudio 1.4.1106.

### Handling missing data

No patient had missing data for Achilles enthesitis at baseline. For the cross-sectional analysis at baseline, linear regression models were conducted using patients with complete data. For the longitudinal analysis, MMRM allowed us to estimate the effects of continuous variables modeling all available data (i.e., using patients with at least one observation during the follow-up). Overall, 70–80% of patients had 2-year follow-up data, while the remaining 20–30% did not reach this follow-up.

## Results

All 749 patients from the REGISPONSER-AS registry were included in this study, since all of them had the required data available concerning the presence of enthesitis. In the overall population, 46 patients (6.1%) had Achilles’ tendon enthesitis detected during the baseline study visit. The majority of the patients in the overall population were male (75.3%), their mean age was 48.4 years old, and 53.1% were smokers (Table 1).

**Table 1:** Baseline characteristics according to the presence of Achilles enthesitis

	**Total** ***N* = 749**	**Current Achilles enthesitis** ***N* = 46**	**No current Achilles enthesitis** ***N* = 703**	***p*-value**
**Sex (male)**	564 (75.3%)	32 (69.6%)	532 (75.7%)	0.352
**Age, mean (SD)**	48.4 (12.3%)	47.8 (10.9)	48.4 (12.4)	0.759
**BMI, mean (SD)**	26.7 (4.3)	27.7 (4.9)	26.6 (4.2)	0.412
**University studies**	91/667 (13.6%)	5/45 (11.1%)	86/622 (13.8%)	0.608
**Single**	109/667 (16.3%)	7/45 (15.6%)	102/622 (16.4%)	0.883
**Smoking (ever)**	267/691 (53.1%)	17/41 (41.5%)	350/650 (53.8%)	0.123
**Age of onset, mean (SD)**	27.1 (10.3)	27.3 (13.2)	27.0 (10.1)	0.709
**Disease duration, mean (SD)**	21.4 (12.7)	20.5 (12.0)	21.5 (12.8)	0.726
**Diagnosis delay, mean (SD)**	8.1 (9.5)	7.6 (8.6)	8.1 (9.6)	0.786
**Family history of SpA**	420/701 (59.9%)	28/40 (70.0%)	392/661 (59.3%)	0.180
**HLA-B27 positive**	586/721 (81.3%)	37 (80.4%)	549/675 (81.3%)	0.880
**Synovitis (ever)**	250/748 (33.4%)	21 (45.7%)	229/702 (32.6%)	0.070
**Psoriasis**	82/746 (11.0%)	7 (15.2%)	75/700 (10.7%)	0.344
**Inflammatory bowel disease**	45 (6.0%)	2 (4.3%)	43 (6.1%)	1.000
**Dactylitis**	55/745 (7.4%)	6 (13.0%)	49/699 (7.0%)	0.141
**Uveitis**	154/745 (20.7%)	8 (17.4%)	146/699 (20.9%)	0.571
**Swollen joints, mean (SD)**	0.3 (1.6)	0.8 (1.7)	0.3 (1.6)	** < 0.001**
**MASES score, mean (SD)**	2.2 (1.9)	2.5 (1.9)	2.1 (1.9)	0.115
**csDMARDs (ever)**	155/742 (20.9%)	27/45 (37.8%)	138/697 (19.8%)	**0.004**
**csDMARDs (current)**	159/746 (21.3%)	18 (39.1%)	141/700 (20.1%)	**0.002**
**Anti-TNF (ever)**	261 (34.8%)	19 (41.3%)	242 (34.4%)	0.343
**Anti-TNF (current)**	157 (21.0%)	10 (21.7%)	147 (20.9%)	0.894
**CRP mg/L, mean (SD)**	9.2 (13.3)	11.0 (14.1)	9.1 (13.2)	0.870
**ASDAS-CRP, mean (SD)**	2.7 (1.1)	3.1 (1.2)	2.7 (1.0)	**0.014**
**ASDAS low disease activity**	206/681 (30.2%)	7/40 (17.5%)	199/641 (31.0%)	0.070
**ASDAS inactive disease**	66/681 (9.7%)	3/40 (7.5%)	63/641 (9.8%)	0.788
**Global VAS (0–10), mean (SD)**	4.6 (2.7)	5.9 (2.6)	4.6 (2.7)	**0.001**
**BASDAI (0–10), mean (SD)**	4.2 (2.4)	5.6 (2.2)	4.1 (2.3)	** < 0.001**
**mBASDAI (0–10), mean (SD)**	4.7 (2.5)	5.7 (2.3)	4.7 (2.5)	**0.007**
**BASFI (0–100), mean (SD)**	38.8 (27.3)	47.3 (27.6)	38.2 (27.2)	**0.031**
**PC-SF12, mean (SD)**	34.5 (11.6)	31.4 (11.8)	34.7 (11.6)	**0.018**
**MC-SF12, mean (SD)**	47.2 (13.8)	46.2 (15.3)	47.3 (13.7)	0.748
**Total BASRI, mean (SD)**	8.0 (4.0)	6.1 (3.0)	7.3 (4.01)	0.068

*Anti-TNF* anti-tumor necrosis factors, *ASDAS-CRP* Ankylosing Spondylitis Disease Activity Score, *BASDAI* Bath Ankylosing Spondylitis Disease Activity Index, *BASFI* Bath Ankylosing Spondylitis Functional Index, *BASRI* Bath Ankylosing Spondylitis Radiology Index, *BMI* body mass index, *global VAS* patient’s global visual analog scale, *CRP* c-reactive protein, *csDMARDs* conventional synthetic disease-modifying antirheumatic drugs, *mBASDAI* modified Bath Ankylosing Spondylitis Disease Activity Index, *MC-SF12* mental component from the SF-12 questionnaire, *NSAIDs* non-steroidal anti-inflammatory drugs, *PC-SF12* physical component from the SF-12 questionnaire, *SD* standard deviation, *SpA* spondyloarthritis

### Profile of patients with Achilles’ tendon enthesitis during the physical examination

Patients with Achilles enthesitis detected during the baseline study visit had similar sociodemographic characteristics (age, sex, study level, and marital status) when compared to patients without Achilles enthesitis (Table 1). Interestingly, no association was found with regard to disease duration, HLA-B27 positivity, psoriasis, IBD, or uveitis. However, patients with Achilles enthesitis showed a significantly higher number of swollen joints during the physical examination than patients without this peripheral symptom (0.8 (1.7) vs. 0.3 (1.6), *p* < 0.001). In addition, patients with current Achilles enthesitis had a more frequent use of previous and current csDMARDs (37.8% vs. 19.8% and 39.1% vs. 20.1%, respectively), although no differences were found concerning anti-TNF intake.

### Influence of the presence of current Achilles enthesitis on PROs

The association between the presence of Achilles enthesitis and the PROs at the baseline visit was evaluated through linear regression (Table 2). Patients with current Achilles enthesitis showed an increase in the global VAS score of 1.31 (95% CI 0.50 to 2.11) points, in the BASDAI (1.47 (95% CI 0.77 to 2.17)), and an increase in the mBASDAI (1.01 (95% CI 0.27 to 1.75)) in comparison with patients without Achilles enthesitis. Similarly, ASDAS-CRP and BASFI scores were increased in patients with current Achilles enthesitis in comparison with patients without this symptom (beta coefficients of 0.44 (95% CI 0.11 to 0.78) and 9.06 (95% CI 0.93 to 17.20), respectively).

**Table 2:** Association between current Achilles enthesitis and patient-reported outcomes at baseline

	**Crude *β* coefficient (95%CI)**^**a**^ **Current Achilles vs. no current Achilles enthesitis**	***β* coefficient (95% CI) adjusted**^**b**^ **Current Achilles vs. no current Achilles enthesitis**
**Global VAS**	**1.31 (0.50 to 2.11)**	**1.45 (0.60 to 2.31)**
**BASDAI**	**1.47 (0.77 to 2.17)**	**1.65 (0.92 to 2.38)**
**mBASDAI**	**1.01 (0.27 to 1.75)**	**1.23 (0.45 to 2.00)**
**ASDAS-CRP**	**0.44 (0.11 to 0.78)**	**0.58 (0.22 to 0.93)**
**BASFI**	**9.06 (0.93 to 17.20)**	**11.58 (3.48 to 19.68)**
**PC-SF12**	− 3.28 (− 6.75 to 0.19)	** − 4.23 (− 7.99 to − 0.47)**
**MC-SF12**	− 1.07 (− 5.20 to 3.05)	− 1.45 (− 5.94 to 3.05)

^a^Linear regression models

^b^Linear regression models adjusted for age, body mass index and current anti-TNF

*ASDAS-CRP* Ankylosing Spondylitis Disease Activity Score, *BASDAI* Bath Ankylosing Spondylitis Disease Activity Index, *BASFI* Bath Ankylosing Spondylitis Functional Index, *Global VAS* patient’s global visual analog scale, *mBASDAI* modified Bath Ankylosing Spondylitis Disease Activity Index, *MC-SF12*, mental component from the SF-12 questionnaire, *PC-SF12* physical component from the SF-12 questionnaire

Since age, BMI, and the current use of anti-TNF therapy may influence both the presence of current Achilles enthesitis and the PROs, additional models adjusted for these variables were explored (Table 2). We found that the increase in global VAS, BASDAI, mBASDAI, ASDAS-CRP, and BASFI scores in patients with Achilles enthesitis remained statistically significant after adjusting for confounders. In addition, a worse physical component score on from the SF-12 questionnaire was observed in patients with current Achilles enthesitis.

### Impact of current Achilles enthesitis on PROs over 2 years of follow-up

Table 3 shows the results of the mixed model with random effects to evaluate the impact of current Achilles enthesitis on PROs after 2 years of follow-up. The mean global VAS, BASDAI, mBASDAI, and ASDAS-CRP scores over the 2 years of follow-up were significantly higher among patients with Achilles enthesitis. After adjusting for age, BMI, and the current intake of anti-TNF, these scores remained significantly higher in these patients.

**Table 3:** Impact of the presence of Achilles enthesitis on patient-reported outcomes over two years of follow-up: mixed models for repeated measures

	**Current Achilles enthesitis** ***N* = 46**	**No current Achilles enthesitis** ***N* = 703**	**Crude *p*-value**	*p*-value adjusted*
**Global VAS, mean (SD)**	5.1 (2.8)	4.3 (2.6)	**0.015**	**0.008**
**BASDAI, mean (SD)**	4.8 (2.7)	3.9 (2.7)	** < 0.001**	** < 0.001**
**mBASDAI, mean (SD)**	5.2 (2.5)	4.5 (2.3)	** < 0.001**	**0.005**
**ASDAS, mean (SD)**	2.9 (1.1)	2.6 (1.0)	**0.007**	**0.001**
**BASFI, mean (SD)**	43.8 (29.7)	40.0 (27.4)	0.245	0.119
**PC-SF12, mean (SD)**	33.3 (11.4)	35.3 (10.7)	0.128	0.064
**MC-SF12, mean (SD)**	47.4 (14.2)	48.2 (12.2)	0.547	0.399

*MMRM* mixed model for repeated measures

^*^Adjusted for age, body mass index and current anti-TNF

*ASDAS-CRP* Ankylosing Spondylitis Disease Activity Score, *BASDAI*, Bath Ankylosing Spondylitis Disease Activity Index, *BASFI* Bath Ankylosing Spondylitis Functional Index, *Global VAS*, patient’s global visual analog scale, *mBASDAI* modified Bath Ankylosing Spondylitis Disease Activity Index, *MC-SF12* mental component from the SF-12 questionnaire, *PC-SF12* physical component from the SF-12 questionnaire

**Fig. 1:**
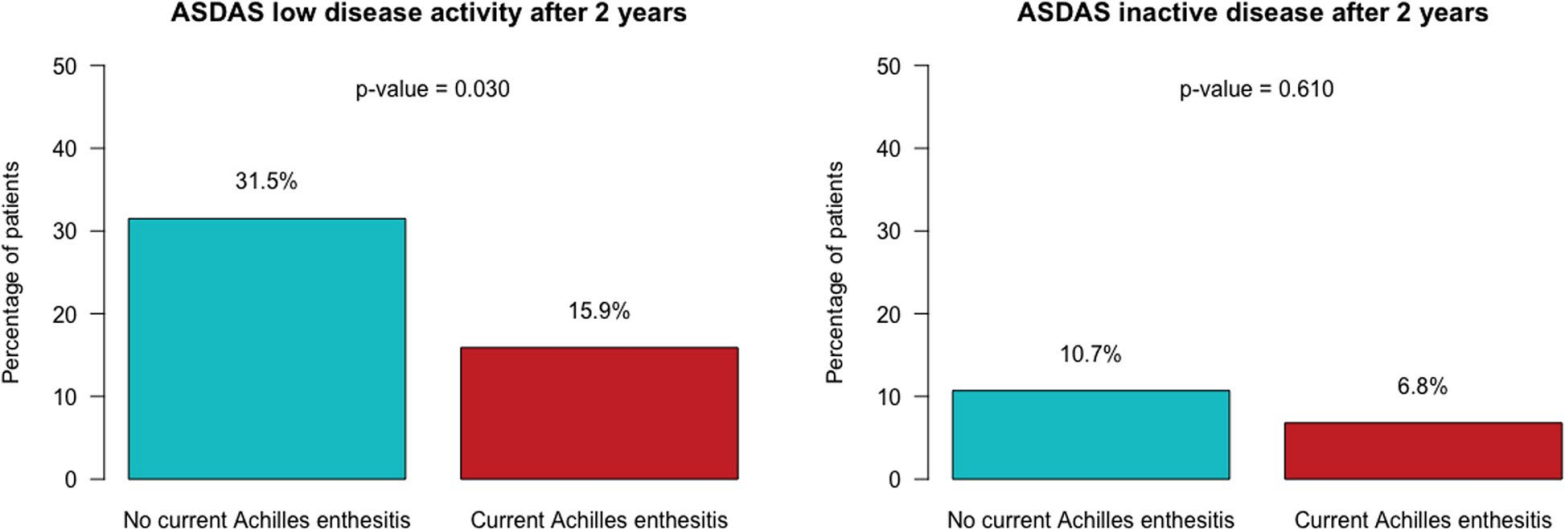
ASDAS low disease activity and ASDAS inactive disease after 2 years of follow-up depending on the presence of Achilles enthesitis on physical examination

We found that the percentage of patients achieving ASDAS low disease activity (ASDAS < 2.1) after 2 years of follow-up was 15.9% and 31.5% for patients with and without Achilles enthesitis, respectively (*p* = 0.030). In addition, 6.8% and 10.7% of patients with and without Achilles enthesitis, respectively, achieved ASDAS inactive disease (ASDAS < 1.3), although these differences were nonsignificant (Fig. 1). Finally, 16.7% of patients with Achilles enthesitis initiated anti-TNF on the next consultation in comparison with 11.3% of patients without Achilles enthesitis. However, these differences were nonsignificant.

### Persistence of Achilles enthesitis after 2 years

Among the 46 patients with Achilles enthesitis at baseline, this symptom persisted after 2 years in 15 (32.6%) patients and was resolved in 31 (67.4%). No specific clinical characteristics associated with the resolution of the enthesitis were found. After 2 years of follow-up, patients with persistent enthesitis were less frequently under anti-TNF therapy (4/15, 26.7%) than patients with resolved Achilles enthesitis (11/31, 35.5%), without significant differences. The anti-TNF agents used by patients with resolved enthesitis were etanercept (7 patients), infliximab (3 patients), and adalimumab (1 patient). Eight patients with resolved enthesitis were under csDMARD monotherapy (either sulfasalazine, methotrexate or leflunomide), and 12 patients did not receive any specific treatment. Concerning patients with unresolved enthesitis, 4 were receiving anti-TNF therapy (2 with etanercept and 2 with adalimumab), one was on sulfasalazine monotherapy, and 10 were without any treatment.

## Discussion

In this prospective study, we found that the presence of Achilles enthesitis during physical examination in patients with AS was associated with higher disease activity and a lower likelihood of achieving low disease activity after 2 years of follow-up in comparison with patients without Achilles enthesitis. To date, many observational studies have evaluated the impact of enthesitis on the burden of disease, but very few have focused on the Achilles tendon. Thus, these results provide new real-life data about this important manifestation in patients with AS.

This study showed that the likelihood of finding Achilles enthesitis in patients with AS during daily clinical practice is 6.1%, and there is no specific profile of patients with a higher probability of suffering from this symptom. Although Achilles enthesitis has been classically associated with psoriatic patients, no association was found with psoriasis in this analysis. This is in line with a previous publication in this same registry showing that, in patients with axSpA, psoriasis was not associated with a history of Achilles enthesitis [20]. On the other hand, these results confirm the relationship between Achilles enthesitis and peripheral synovitis during physical examination. Although enthesitis can occur at sites distant from the joints (such as the Achilles tendon and plantar fascia), enthesitis is usually periarticular, which can lead to secondary synovitis [5]. In addition, because entheses are found in direct conjunction with the joints, sometimes the peripheral pain in these patients might come from enthesitis rather than from synovitis. Thus, the attribution of joint pain to synovitis can lead to an underestimation of the prevalence of enthesitis [5, 21].

Since the Achilles tendon and plantar fascia are located in the lower limbs, these regions are exposed to higher mechanical forces during daily activities, and this may have an adverse impact on the patient’s daily life and have socioeconomic consequences. In fact, we found that patients with Achilles enthesitis showed worse function as measured with the BASFI in comparison with patients without Achilles enthesitis, as well as higher disease activity. However, it should be noted that this impact on PROs is not limited to a particular visit in which Achilles enthesitis is present, but the impact is maintained over 2 years. Our results suggest that patients with this symptom at baseline during physical examination had worse global assessment scores and higher disease activity (as measured by the BASDAI, mBASDAI, and ASDAS) after 2 years of follow-up after adjusting for the treatment, demonstrating that patients that maintained higher scores on PROs had a lower likelihood of achieving low disease activity or inactive disease. Interestingly, the mBASDAI (which omits the 3rd and 4th questions of the BASDAI) was persistently higher over the 2 years of follow-up in the group with Achilles enthesitis, suggesting that this feature is also associated with higher levels of fatigue, axial pain, and stiffness. On the other hand, we found a significant effect of Achilles enthesitis on ASDAS and ASDAS low disease activity, which include questions about peripheral symptoms. These results confirm that Achilles enthesitis influences outcomes related not only to peripheral features but also to other disease-related parameters. However, although this symptom could be considered a poor prognostic factor, it does not appear to influence the initiation of anti-TNF.

After 2 years of follow-up, we found that Achilles enthesitis persisted in 32.6% of patients and was resolved in 67.4%, with a slightly nonsignificant greater use of anti-TNF in the second group. This finding does not mean causality or potential efficacy of anti-TNF in treating this symptom, since this is an observational nonrandomized prospective analysis in which a prescription bias may be present.

Overall, these results highlight the necessity of treating and controlling heel enthesitis in these patients. Many observational and randomized clinical trials (RCTs) have evaluated the effect of IL-17, IL-23, TNF, and JAK inhibition on the resolution of enthesitis. However, the accumulated knowledge comes from post hoc or secondary analyses using enthesitis scores as endpoints, without focusing on the heel [22–26]. To date, only two RCTs have focused on the resolution of heel enthesitis as the primary outcome. The first one was the HEEL trial, a 12-week, randomized, double-blind, placebo-controlled study that compared etanercept with placebo in patients with heel enthesitis confirmed by magnetic resonance imaging (MRI) [27]. This study demonstrated a significantly greater reduction in the patient’s global assessment of heel disease activity for etanercept in comparison with the placebo group. The second one was the ACHILLES study, a 52-week, randomized, double-blind, placebo-controlled study in patients with PsA and axSpA with MRIs positive for heel enthesitis that compared secukinumab vs. placebo [28]. In this case, the primary endpoint (superiority of secukinumab over placebo based on the percentage of patients with clinical resolution of Achilles’ tendon enthesitis as assessed by the respective subcomponent of the Leeds Enthesitis Index (LEI) at week 24) was not met. These limited results and the relevance of this symptom raise the need to propose future observational studies and RCTs focused on this feature as the primary outcome.

This study has some limitations and strengths. One limitation is that the Achilles enthesitis was not confirmed with ultrasound, which has been demonstrated to be useful in daily clinical practice to evaluate the presence of inflammatory and structural lesions [29]. It should be noted that this registry was launched between 2004 and 2007, by which date the implementation of ultrasound in rheumatology clinics was not complete. Another limitation is the absence of information on concomitant fibromyalgia, which can be associated with a higher prevalence of enthesitis and tender points in SpA patients [30]. We acknowledge that a risk of misclassification of patients with enthesitis may exist in this context. This could have implications, especially in the results after 2 years, when patients may have other reasons for a tender Achilles tendon, such as fibromyalgia or mechanical problems. The low frequency of anti-TNF treatment in patients with persistent enthesitis might be related to these findings. However, investigators participating in the REGISPONSER study were rheumatologists who were experts in the field of SpA and were able to correctly identify clinical Achilles enthesitis. The last limitation was the fact that the presence of bilateral enthesitis was not possible to determine due to the data collection scheme in this registry.

One strength of this study is the homogeneity of the population, since all of these patients had a confirmed diagnosis of AS. Another strength is that additional models adjusted for age, BMI, and anti-TNF intake were explored since these variables may influence both the presence of Achilles enthesitis and the PROs. Finally, the use of “current” Achilles enthesitis rather than Achilles enthesitis “ever” avoided recall bias by the patients.

## Conclusions

In summary, these results showed the impact of the presence of Achilles enthesitis on the burden of the disease (not only at the moment of the visit but also after 2 years of follow-up), leading to a lower probability of achieving low disease activity. This study confirms the relevance of Achilles enthesitis in patients presenting with AS, emphasizing the importance of a systematic, iterative check for this clinical feature during the monitoring of these patients.

## Data Availability

The datasets generated and/or analyzed during the current study are not publicly available but are available from the corresponding author on reasonable request.

## Abbreviations

Anti-TNF: Anti-tumor necrosis factor
AS: Ankylosing spondylitis
ASAS: Assessment of Spondyloarthritis International Society
ASDAS-CRP: Ankylosing Spondylitis Disease Activity Score
AxSpA: Axial spondyloarthritis
BASDAI: Bath Ankylosing Spondylitis Disease Activity Index
BASFI: Bath Ankylosing Spondylitis Functional Index
BASRI: Bath Ankylosing Spondylitis Radiology Index
bDMAiRDs: Biological disease-modifying antirheumatic drugs
BMI: Body mass index
CRP: C-reactive protein
csDMARDs: Conventional synthetic disease-modifying antirheumatic drugs
ESSG: European Spondylarthropathy Study Group
global VAS: Patient’s global visual analog scale
IBD: Inflammatory bowel disease
mBASDAI: Modified Bath Ankylosing Spondylitis Disease Activity Index
MASES: Maastricht Ankylosing Spondylitis Score
MC-SF12: Mental component from the SF-12 questionnaire
MEI: Mander Enthesitis Index
MMRM: Mixed model for repeated measures
NSAIDs: Non-steroidal anti-inflammatory drugs
PC-SF12: Physical component from the SF-12 questionnaire
PROs: Patient-reported outcomes
REGISPONSER: Spondyloarthritis Registry of the Spanish Rheumatology
SD: Standard deviation
SpA: Spondyloarthritis
